# Expression and distribution of neuroglobin and hypoxia‐inducible factor‐1α in the adult yak telencephalon

**DOI:** 10.1002/vms3.553

**Published:** 2021-06-19

**Authors:** Xiaohua Du, James Blackar Mawolo, Xia Liu, Xiaoyu Mi, Qiao Li, Yongqiang Wen

**Affiliations:** ^1^ College of Veterinary Medicine Gansu Agricultural University Lanzhou City Gansu Province People's Republic of China; ^2^ College of Life Science and Technology Gansu Agricultural University Lanzhou City Gansu Province People's Republic of China

**Keywords:** Hif ‐1a, Ngb, oxygen, telencephalon, yak

## Abstract

The telencephalon is also known as the cerebrum, and it consists of the largest part of the brain. It makes up about 85% of the total weight of the brain. Neuroglobin (Ngb) is a protein found in neurons of both the peripheral and central nervous system that appears to convey some resilience to hypoxia, while the hypoxia‐inducible factor (Hif‐1α) is a dimeric protein complex that plays an integral role in the body's response to low oxygen concentrations, or hypoxia. The study examines the expression of Ngb and Hif‐1α in the telencephalon of adult yak in the telencephalon. The immunohistochemistry (IHC), quantitative real‐time PCR and Western blot (WB) were employed to investigate Ngb and Hif‐1α expression in the telencephalon. Ngb and Hif‐1α are significantly expressed in all tissues of the telencephalon except the hypothalamus. The cerebellar cortex, hippocampus, amygdala, cerebellum and corpus callosum recorded the highest expression but not significant. The overall expression revealed that Ngb expression was higher as compared to Hif‐1α. The IHC results also showed that the expression of Ngb and Hif‐1α were higher in the cerebellar cortex, hippocampus, amygdala, cerebellum and corpus callosum as compared to other regions. The results suggested that Ngb and Hif‐1α expression influence the adaptive mechanism of yak to the high altitude environment. Both Ngb and Hif‐1α participate in oxygen transports throughout the telencephalon and have functions in neuroprotection. Further studies are needed to confirm the mechanism of adaptation.

## Graphical abstract

The study examines the expression of Ngb and Hif‐1α in the telencephalon of adult yak and explores the factors causing the different levels of Ngb and Hif‐1α in the telencephalon.

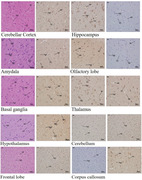

## 1 INTRODUCTION

Altitude is a component of the physical environment to which animals show adjustments (Hall et al., 1963). High‐altitude environments present many physiological challenges for mammals. Indeed, because the increasing altitude is characterized by decreasing oxygen availability in the environment without major changes in tissue requirements. The telencephalon which is the largest part of the brain contains the cerebral cortex (of the two cerebral hemispheres), as well as several subcortical structures, including the hippocampus, basal ganglia and olfactory bulb. In the yak, it begins to emerge in embryonic development at about 5 weeks. At this time, the nervous system consists of a tube‐shaped piece of tissue called the neural tube which later develops into important structures in the nervous system. At the start of 2000, Burmester et al. observed that Ngb is expressed in the vertebrate nervous system especially occupying the central and peripheral nervous system (CNS and PNS), (Burmester et al., 2000). Ngb is a hypoxia‐inducible protein found in both peripheral and central nervous systems that appears to convey some resilience to hypoxic/ischemic insult, perhaps by facilitating oxygen transport across the blood‐brain barrier or enhancing the availability of oxygen to mitochondria. Zhao et al. reported that Ngb is highly expressed in the brains of mice with traumatic brain injury (Zhao et al., 2012) while in the hypothalamus, amygdala, and pontine tegmental nuclei of humans, Ngb was significantly expressed (Hundahl et al., 2013). In the pig brain, Ngb levels in the hypothalamus were higher than the frontal cortex. The lowest difference was found in sheep, which showed Ngb expression in the hypothalamus and cerebrum (Fabrizius et al., 2016). Oxygen (O_2_) is vital for respiration, which is the process that transfers energy from glucose to cells. In various physiological and biochemical states, organisms encounter limited O_2_ availability or hypoxia. To adapt to this condition, evolutionarily conserved responses must be engaged. In adult mammals and other non‐vertebrate species, the primary transcriptional factor that responds to hypoxic stress is mediated by a dimeric protein called the Hif‐1α. Hif‐1α is an important transcription factor that regulates oxygen consumption and morphologically changes in response to varying oxygen concentrations. Frank et al. reported that Hif‐1α targets genes in the adult rat brain and promotes cell survival in hypoxic tissues (Frank et al., 2001). A study conducted by Jaona et al. ascertained that Hif‐1α expression in the hypothalamus regulates cellular responses to inflammation and hypoxia, being essential for normal cell function and survival (Jaona et al., 2018). Despite these reported references, a connection between Ngb and Hif‐1α has not been demonstrated directly, and some evidence argues against it. First, the Ngb promoter region appears to lack consensus Hif‐1α ‐binding hypoxia‐response elements (Wystub et al., 2004). Although few researchers have recorded data about Ngb and Hif‐1α expression, however, none of them have focused on the Ngb and Hif‐1α connection, exact function, pattern, quantities of expression, and the mechanism is still a matter of debate among thinkers. Therefore; the present study aims to provide detailed references about the connection of Ngb and Hif‐1α expression in the telencephalon of adults and further investigated the factors causing the diverse pattern and quantity of expression. The adult yak is a long‐haired domesticated bovid found throughout the Himalayan region of the Indian subcontinent, north Mongolia and the Tibetan Plateau. It plays an important role in the economic and life activities of people living in the Qinghai‐Tibetan plateau and nearing mountainous areas. The yak is also involved in the cultural and social life of the herders and their families. The research provided essential morphological, physiological and biochemical data about the relationship between Ngb and Hif‐1α expression. In addition, the results contribute to the advancement of Ngb and Hif‐1α expression and elaborated on the adaptive mechanism.

## 2 MATERIALS AND METHODS

### 2.1 Animals and setting

The Animal Ethics and Welfare Committee of Gansu Agricultural University in October of 2019 (AEWC‐GAU‐2019‐042) reviewed and approved all experimental procedures performed in this study. All animals were housed in a full facility at the Hezuo Xingfa Yak and Sheep Breeding Cooperation Center in the Gannan Tibetan Autonomous Prefecture in Gansu Province of China. Three (3) healthy adult yaks at the age of 3 years were purchased from the centre. The animals were housed and monitored by trained personnel and fed on grasses and sedges, such as Carex, Stipa and Kobresia. In the plateau environment of Gannan Tibetan Autonomous Prefecture, the altitude was 3000 m. Experiments were carried out using adult yak weighing 550–720 kg. The weight ranges from 10 to 15μm. The animals were maintained at a temperature between ‐7° C and ‐8° C and had free access to food and water. Every effort was applied to reduce the number of animals used and minimize animal suffering during the sampling process.

#### 2.1.1 Treatment and specimen techniques

Animals were retrieved one at a time from their living areas and minimally immobilized to facilitate sacrificing and then extraction of the brain. As per the guidance of resident veterinarians, this practice was undertaken to reduce harm and pain to the animals. Upon sacrificing each animal, the whole brain was quickly extracted by craniotomy. Subsequently, the cerebral cortex, frontal lobe and temporal lobe among others were extracted. Tissue samples prepared for immunohistochemistry were fixed in 4% paraformaldehyde (PH 7.4, w/v) and samples for quantitative reverse transcription‐polymerase chain reaction (qRT‐PCR) and western‐blotting were stored at ‐80°C.

#### 2.1.2 Reagents and instrumentations

Quantitative real‐time polymerase chain reaction (qRT‐PCR) reagents and supplies are AG RNAex Pro RNA kit, SYBR Green Pro Taq HS kit, Evo M‐MLV reverse‐transcription kit (removal gDNA reagent) and Rox and were purchased from Accurate Biotechnology (Hunan) Co. Ltd. People's Republic of China. Western‐blotting reagents and supplies are Rabbit Anti‐Ngb, Polyclonal Antibody (bs‐1859R), Rabbit Anti‐HIF‐1, Alpha Polyclonal Antibody (bs‐0737R), Rabbit Anti‐beta‐Actin (Loading Control), Polyclonal antibody (bs‐0737R) and goat anti‐rabbit IgG/HRP(bs‐0295G‐HRP) and were purchased from Bioss Co. Ltd. People's Republic of China. RIPA tissue or cell rapid lysate was purchased from Bio topped, and 0.22μm polyvinylidene difluoride filter (PVDF) membranes, 4 × protein loading buffer (DTT), Rainbow 245 broad‐spectrum protein marker (11‐245KD) and ECL hypersensitivity luminescent solution were purchased from Solarbio Co. Ltd. People's Republic of China. Immunohistochemical reagents and supplies are immunohistochemical staining kit and HRP‐DAB kit and were purchased from Beijing Zhongshan Golden Bridge Biotechnology Co. Ltd. People's Republic of China.

#### 2.1.3 Total RNA isolation and qRT‐PCR

Total RNA was isolated using the TRIzol reagent (Accurate Biotechnology, China). Eight hundred nanograms of total RNA were reverse transcribed using the Evo M‐MLV cDNA synthesis kit (Accurate Biotechnology, China). Real‐time PCR was performed using Quant Studio 5. The qRT‐PCR primer sequences and accession numbers are shown in Table 1. Reaction mixtures (20 μl) consisted of 10 μL SYBR Green Pro Taq (Accurate Biotechnology, China), 0.8 μl forward and reverse primers (0.2 μmol/ml), 0.4 μl Rox, 2 μl cDNA, 6 μl ddH2O. The thermocycler was set to 50°C 2 min, 95°C 2 min, 40 cycles at 95°C 10s, annealing for 34 s (annealing temperatures are shown in Table 1), with melting temperatures examined from 65°C to 95°C, increments of 0.5°C every 5 s. The 2^−ΔΔCt^ method was used to analyze the expression of Ngb and Hif‐1α mRNA relative to β‐actin mRNA expression according to the system‐generated Ct value.

**TABLE 1:** Primer sequences of target and house‐keeping genes

Primer name	Accession numbers	Sequence (5′ to 3′)	Tm/°C	Amplicon size	Note
Ngb	JQ241373.1	F:CTTTCGCCAGGCTGTTTGA	60.0	134	qRT‐PCR
		R:CTGATGTGGTCCAGGAACTCG			
HIF‐1α	NM_174339.3	F:CTACATTACCTGCCTCTGAAACTCC	59.8	146	qRT‐PCR
		R:ACGCTTTGTCTGGTGCTTCC			
β‐actin	NM_173979.3	F: ATATTGCTGCGCTCGTGGT	60.2	158	qRT‐PCR
		R: TCATCCCCCACGTACGAGTC			

#### 2.1.4 Western‐blotting

For Western blotting analyses (Song et al., 2018), frozen tissue samples from different regions were weighed. After that, the tissues were homogenized using a glass rod in lysis buffer (1 ml RIPA + 10 μl PMSF) at ice‐cold temperature, shaken in an ice bath for 2 h (120r /min), and centrifuged at 12,000 rpm at 4°C for 10 min to collect the supernatant. The protein was subjected to SDS polyacrylamide gel electrophoresis. Separated proteins were transferred to a polyvinylidene difluoride filter (PVDF) membrane via the transfer apparatus at 110 V for 60 min. The membranes were then blocked via 5% milk/PBST at 4°C overnight and then incubated with primary antibody against Ngb, Hif‐1α and β‐actin for 3 h at room temperature. The antibody concentrations (v/v) of Ngb, Hif‐1α and β‐actin were 1:800, 1:500, 1:3000. The membranes were washed thrice (10 min each) with PBST and incubated with secondary antibody (HRP‐conjugated goat anti‐rabbit IgG, 1:4000) for 1 h at room temperature. After thrice washing in PBST (10 min each), the membranes were scanned with the ECL western‐blotting machine (GE AI600, America). Each group of protein was repeated three times. The signals were analyzed with Image J software (NIH, Bethesda, MD, USA) to determine the relative expression levels of Ngb and Hif‐1α.

#### 2.1.5 Immunohistochemical staining

Tissue samples from the telencephalon of the adult yak brain were fixed (4% paraformaldehyde) and trimmed (2 cm × 2 cm). Then, the tissue samples used conventional gradient alcohol dehydration, made tissue wax blocks with paraffin embedding, cutting tissues with serial sections (thickness 4 μm), exhibiting, patching, baking sheet processing, hematoxylin‐eosin (HE) routine staining, microscopy. The paraffin‐embedded tissue sections were deparaffinized in xylene and then rehydrated in graded alcohol. The PBS (0.01 mol/L, pH = 7.2) was rinsed three times, each time 5 min. Note that 0.125% trypsin antigen was repaired 30 min and rinsed in PBS two times. The endogenous peroxidase activity was blocked by incubating the sections for 10 min in 30 ml/L hydrogen peroxide blocking solution, followed by rinsing three times with PBS for 5 min each time to reduce non‐specific binding of the first antibody. Normal sheep serum was added for blocking and incubated at room temperature for 15 min. The corresponding primary antibody was added in the sections, incubated at 37°C for 2 h and rinsed in PBS three times. The appropriate secondary antibody was added after being removed from PBS and incubated at 37°C for 15 min. The streptomyces avidin‐peroxidase solution was added in the sections, incubated at 37°C for 15 min, PBS was rinsed three times, 5 min each time. The immuno‐peroxidase color reaction was developed with the HRP‐DAB substrate chromogen solution after removed PBS. Distilled water stopped the reaction, and the sections were lightly counterstained with hematoxylin, dehydrated, in increasing concentrations of ethanol, cleared and covered with mounting medium and coverslips (at 4°C). Then the sections were stored at 20°C until used for taking photographs and microscopic analysis. To assess the specificity of the immunolabelling, the negative controls were performed by substituting the primary antibody with PBS. Other procedures remained constant.

#### 2.1.6 Anesthesia procedures

As per the regulations of the Animal Ethics and Welfare Committee of Gansu Agricultural University, all animals involved in the study were placed separately until they were confirmed to be healthy. The animals were observed for 2 weeks before further procedures were performed according to the committee regulations. Animals were free while under observation, and the observation confirmed that the animals were free of specified infectious diseases that could have an adverse effect on the experimental procedures. The animals' diet was provided while under observation and no search for food, chewing, grubbing and gnawing during the activity periods. To avoid or minimize pain, the animals were treated calmly by trained personnel. Dealing with these large animals requires more personnel, so additional trained personnel were employed for assistance during the sacrifice. The animals were spoken to by the personnel, and loud sounds were avoided to avoid the animals escaping. More food was simultaneously given to the animals to enable interaction between the animals and the personnel and supported a developing relationship with the personnel. The animals were made to lie on their side by scratching their back and flanks by the personnel. While in a calm state, the injections were administrated and scarification took place. The injections were performed under slow pressure to reduce pain and careful extraction of the samples. The injection needles were appropriate for the animal size. The environment was well lit, and sharp or damaged objects were removed.

#### 2.1.7 Animals housing conditions

The Hezuo Xingfa Yak and sheep breeding cooperation are located in Hezuo city, Gansu Province, the People's Republic of China. With an elevation of nearly 3000 meters (9,800 ft), Hezuo has an alpine subarctic climate, with long, very cold, dry winters, and short, mild summers. The monthly daily mean temperature in January, the coldest month, is −9.3°C (15.3°F), while the same figure for July, the warmest month, is 13.3°C (55.9°F); the annual mean is 2.82°C (37.1°F). Most of the annual precipitation is delivered from May to September. With monthly percent possible sunshine ranging from 44% in June and September to 71% in December, the city receives 2370 h of bright sunshine annually. The animals live together (male and female) to enable reproduction. Yaks are directed to the calving area before calving. The veterinarians monitor the feeding habitat and health status of the animals at the center. The animals are trained to cooperate with the veterinarians and investigative personnel. When it rains, the animals enter the chutes or cages with the aid of the centre guides. The centre is surrounded by a fence, and security measures are employed to safeguard the animals.

#### 2.1.8 Sacrifice

A jugular intravenous injection was applied to perform the procedure which requires at least two individuals. One to restrain the animal while the other performed the injection. An appropriately sized needle was used. The syringe was drawn up with the required dose, and all air bubbles were expelled. The clip fleece from the side of the animal's neck which showed the jugular vein was exposed, and the needles or catheters were inserted at an angle of approx. 10–20° into the animal's neck. Blood was gently drawn in the hub of the needle and flowed freely into the syringe. Subsequently, the desired dose was injected. The personnel withdrew the needle and applied pressure until bleeding ceased.

#### 2.1.9 Euthanasia method

Barbiturates and barbituric acid derivatives were employed because of their rapid action and ability to induce a smooth transition from consciousness to unconsciousness and death. Despite its drawbacks which are cost‐intensive and need to restraint the drug, the necessity to maintain a careful accounting of amounts used, requirements that these agents were administered by trained personnel who are registered with the Gansu Province Drug Enforcement Administration, and finally, residues that limit carcass disposal options. The general endorsement of the use of intravenous injection of T‐61, barbiturates, mixture with succinylcholine mixed in a 10: 1 to 20: 1 ratio was applied. The adult yaks tend to move rear and go over backward when given any of these injectable compounds, particularly barbiturates are administrated. This type of event naturally threatens everyone near the horse, but particularly the operator. These yaks were controlled by adding succinylcholine to the solution to avoid using heavy tranquilization before euthanasia.

### 2.2 Data analysis

Statistical analyses were performed using SPSS version 22 (SPSS, Inc., Chicago, IL, USA). The data for Ngb and Hif‐1α protein levels were subjected to analysis of variance (ANOVA), and the treatment means were separated by Duncan's multiple range test at (*p* < 0.05) using SPSS 22.0 version. Data were presented as mean and standard deviation (SD). Statistical significance was defined as *p* < 0.05. The expression intensity was analyzed using Image J software and calculated according to the software standard value.

## 3 RESULTS AND DISCUSSION

### 3.1 Determination of results

Immunohistochemical (IHC), Real‐time PCR (qRT‐PCR) and Western Blot (WB)

An immunohistochemical study was performed to assess the specific localization of Ngb and Hif‐1α between tissues of the adult yak telencephalon. Real‐time PCR for Ngb and Hif‐1α mRNA levels, quantification of Ngb levels in the telencephalon of adult yak. The analysis is presented in Table 2, which includes the mean ± standard deviation, minimum and maximum level, percentage and significance. Western blot was performed to detect and confirm the protein levels of Ngb and Hif‐1α in the telencephalon tissues of the adult yak.

**TABLE 2:** Ngb and Hif‐1α expression in the telencephalon tissues of adult yak

Tissues	Factors	Mean ± SD	Minimum	Maximum	%	Significant rate
Cerebellar Cortex	Ngb	12.179 ± 0.150	11.805	12.553	63.1%	0.000***
	Hif‐1α	5.276 ± 0.015	5.238	5.314	36.9%	0.000***
Hippocampus	Ngb	11.538 ± 0.118	11.243	11.832	65.1%	0.002**
	Hif‐1α	6.174 ± 0.043	6.065	6.282	34.9%	0.002**
Amygdala	Ngb	11.125 ± 0.470	10.593	11.483	66.1%	0.003**
	Hif‐1α	5.712 ± 1.416	4.081	6.635	33.9%	0.003**
Olfactory lobe	Ngb	10.690 ± 0.321	10.339	10.970	66.7%	0.000***
	Hif‐1α	5.346 ± 0.321	4.992	5.621	33.3%	0.000***
Basal ganglia	Ngb	11.022 ± 0.152	10.644	11.400	66.3%	0.015**
	Hif‐1α	5.595 ± 0.118	5.300	5.891	33.3%	0.015**
Thalamus	Ngb	10.884 ± 0.108	10.729	10.968	66.2%	0.008**
	Hif‐1α	5.551 ± 0.094	5.461	5.640	33.8%	0.008**
Hypothalamus	Ngb	11.134 ± 0.043	11.097	11.182	64.1%	0.210
	Hif‐1α	6.238 ± 0.013	6.229	6.254	35.9%	0.210
Cerebellum	Ngb	11.805 ± 0.212	11.278	12.331	67.2%	0.000***
	Hif‐1α	5.232 ± 0.059	5.084	5.379	35.3%	0.000***
Frontal lobe	Ngb	10.707 ± 0.065	10.544	10.869	67.2%	0.014**
	Hif‐1α	5.214 ± 0.142	4.860	5.568	32.8%	0.014**
Corpus Callosum	Ngb	11.961 ± 0.008	11.941	11.984	63.1 %	0.000***
	Hif‐1α	7.009 ± 0.028	6.939	7.079	36.9 %	0.000***

Ngb and a Hif‐1α were significantly expressed in the cerebellar cortex, hippocampus, amygdala, cerebellum, while other regions demonstrated fewer expressions.

#### 3.1.1 Results description

The descriptive statistics for the expression of Ngb and Hif‐1α in the adult yak are presented in a table and graphs. The Immunohistochemical results are reported in images. Table 1 showed the primer sequence, while Table 2 presented a full analysis of the Ngb and Hif‐1α expression in the adult yak brain tissues, and Figures 1a and 1b compared the Ngb and Hif‐1α expression in the adult yak. The results reported that Ngb and Hif‐1α were significantly expressed in several brain tissues for the adult yak, while others were higher but no significant. The significant and higher expressions play an important function in the adaptation of the yak to the high altitude environment. Figures 2a and 2b recorded the immunohistochemical results in all brain tissues mentioned in the study. The western blot results are placed appropriately. The trends of Ngb and Hif‐1α expression were widely distributed in the brain tissues of the adult yak. Ngb and Hif‐1α were significantly expressed in the cerebellar cortex, hippocampus, amygdala, cerebellum and corpus callosum, while other regions demonstrated less expression. However, it was recorded that the hypothalamus showed higher but without significance. The overall expressions of Ngb were higher than.

FIGURE 1(**a)** The expression of Ngb and Hif‐1α in the telencephalon of adult yak. The comparison reveals that Ngb is highly expressed in the cerebellar cortex, hippocampus and amygdala as compared to Hif‐1α. However, Hif‐1α is found higher in the hippocampus than other tissue regions. Ngb overall expression is higher than Hif‐1α. (**b)** The expression of Ngb and Hif‐1α in the telencephalon of adult yak. The corpus callosum and cerebellum were reported higher as compared to Hif‐1α. Ngb overall expression is higher than Hif‐1α

**Figure:**
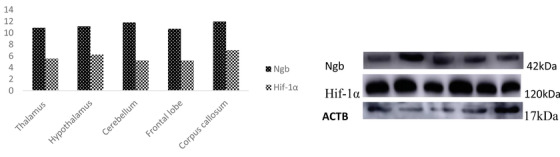

**Figure:**
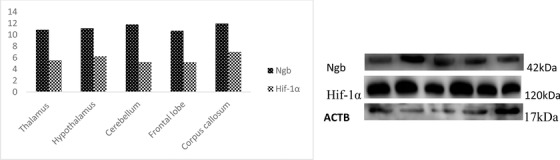

FIGURE 2Ngb and Hif‐1α expression in the telencephalon of adult yak. (a) The expression of Ngb and Hif‐1α in the cerebellar cortex of young yak diencephalon. Positive and negative controls are indicated by arrows. The expression is shown in the middle region of this tissue. (b) Ngb and Hif‐1α are found in the lower regions of the hippocampus. (c) Ngb is observed in the upper region of the amygdala while Hif‐1α was located in the middle right extreme of the amygdala. (d) Ngb is found in the middle region of the olfactory lobe and Hif‐1α in the same region. (e) Ngb as seem is located in the upper region of the basal ganglia, while Hif‐1α is observed in the upper region. (f) Ngb is heavily found in the entire region of the thalamus, and Hif‐1α is shown in the upper and lower regions. (g) Ngb and Hif‐1α are found in the middle region of the cerebrum. (h) Ngb as seem is located in the upper region of the hypothalamus, while Hif‐1α is observed in the upper region. (i) Ngb is observed in the middle region of the cerebrum, while Hif‐1α was located in the lower right extreme of the amydala. (j) Ngb and Hif‐1α are found in the lower and middle regions of the frontal lobe, respectively. (k) Ngb is observed in the middle region of the corpus callosum and Hif‐1α in the same region

**Figure:**
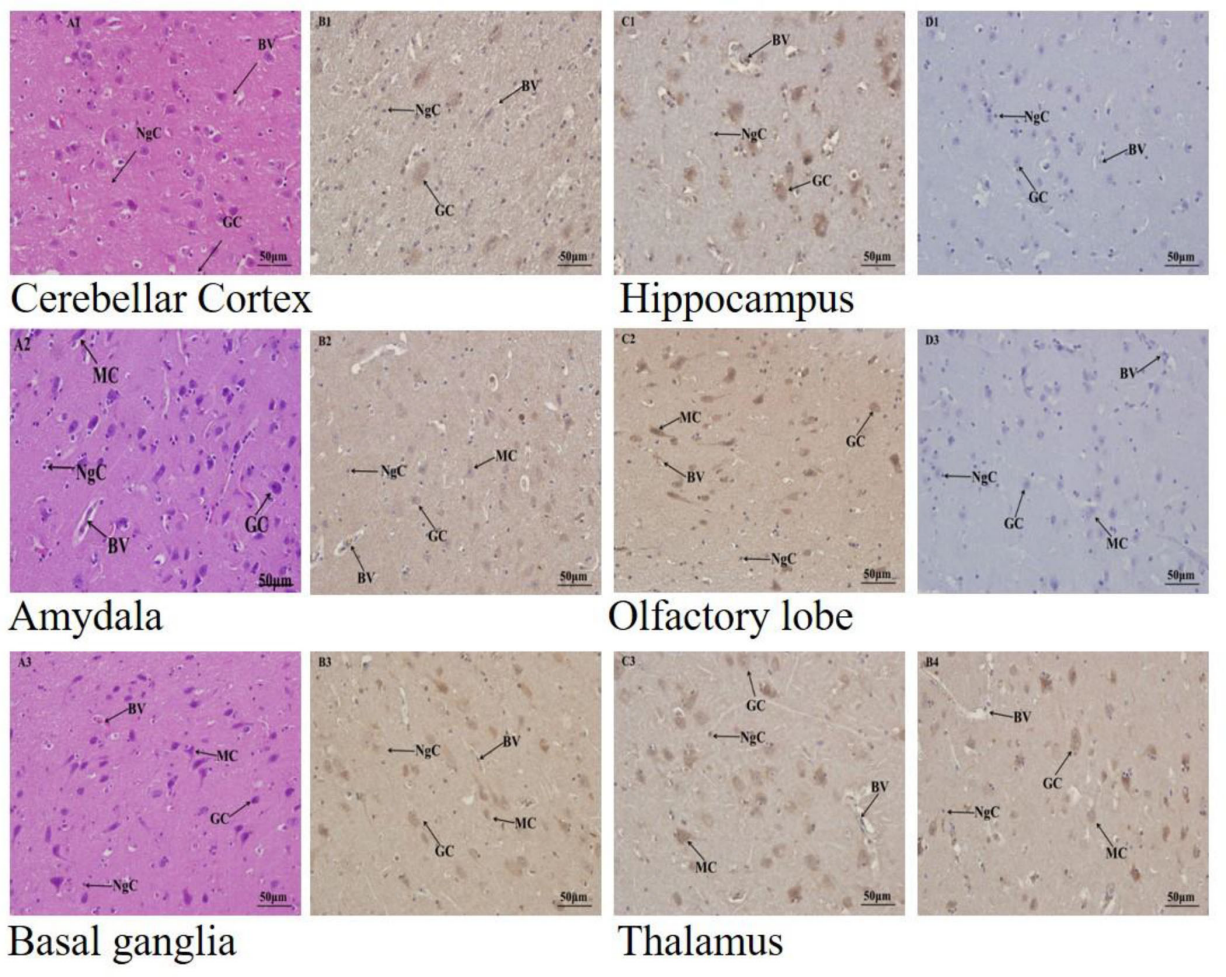

**Figure:**
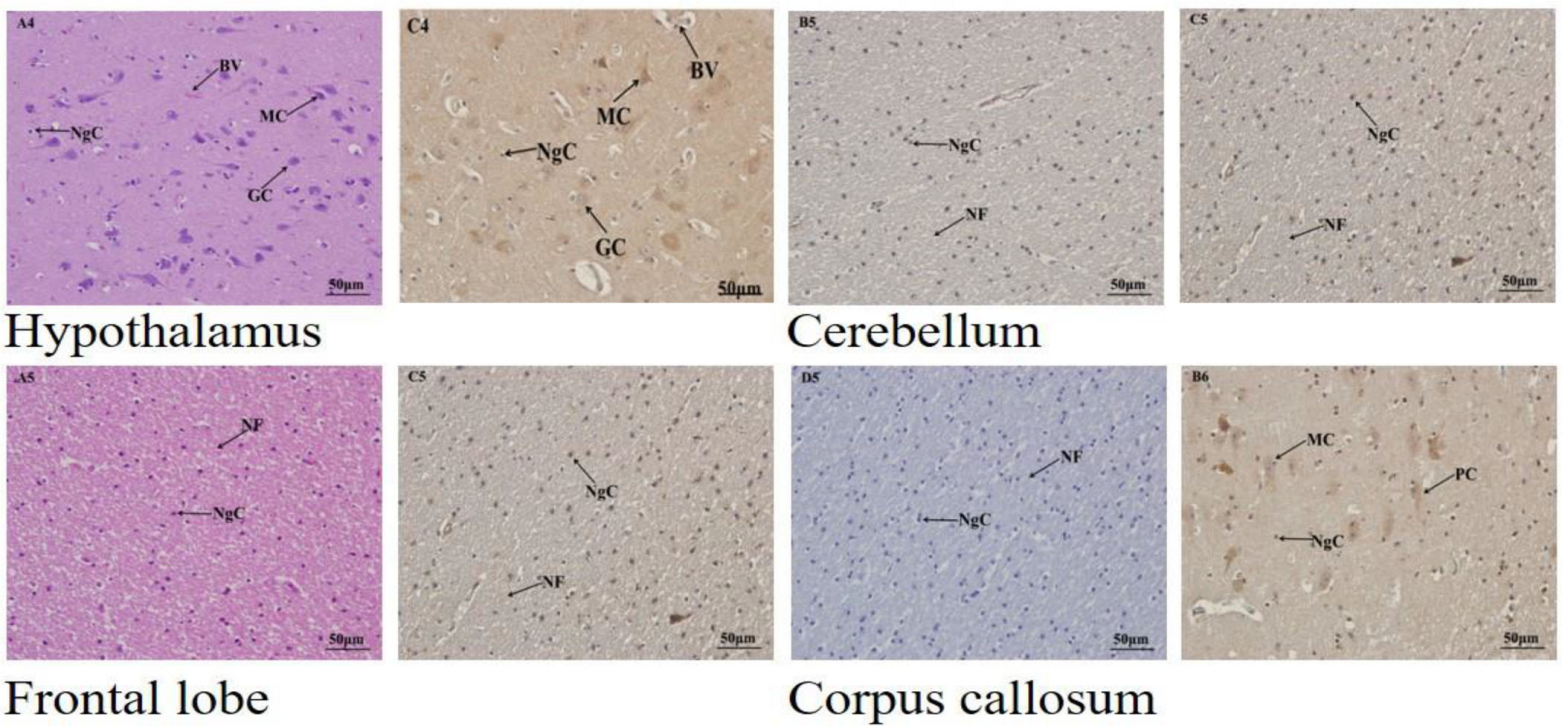

## 4 DISCUSSION

The pattern of Ngb and Hif‐1α expression varies in different tissues of the telencephalon, and factors leading to the different expression have gone unnoticed. Besides, the results provided a vivid explanation about oxygen function in the neuronal tissues relating to the adaptive mechanism of adult yak.

### 4.1 Cerebellar cortex

The cerebellar cortex has a role in receiving information from most regions of the body and alerts the brain. According to Reuss et al. Ngb is solely expressed in the cerebellar cortex of the rodent brain. Purkinje cells of the cerebellar cortex also showed a level of Ngb mRNA expression (Reuss et al., 2002). A study performed by Christian et al. also confirmed the significant Ngb expression in the cerebellar cortex of the adult mouse brain, but Fabrizius et al. interestingly revealed a lower Ngb expression in the cerebellar cortex during fetal development of the mouse brain and has a tendency to increase as the mouse approaches adulthood (Fabrizius et al., 2016). The current study displayed a significant level of Ngb and Hif‐1α expression in the cerebellar cortex of adult yak, but Ngb was highly expressed than Hif‐1α. Despite its higher expression, Hif‐1α regulates Ngb expression in the channels of information from other tissues of the telencephalon and activates Ngb, while responding to the signal. Ngb plays a protective role in the control movement and influences many other functions in the cerebellar cortex, and Hif‐1α activates or modifies Ngb expression. In the human brain, a low Ngb expression was reported (Hundahl et al., 2013), while Huquing et al. reported that Hif‐1α expression increased with increasing age in the cerebellar cortex between 3 and 18 months (Huquing et al., 2013).

### 4.2 Hippocampus

Hippocampus responds to emotional stimulation. In the study of vertebrae globin expression, Burmester et al. reported that Ngb is expressed at 11% in the hippocampus of the human brain while finding reported by Reuss et al. recorded positive expression of Ngb is involved in the formation of the rodent's hippocampus (Burmester et al., 2000; Reuss et al., 2002). The present findings revealed a significant level of Ngb and Hif‐1α levels in the hippocampus of the adult yak. However, the expression of Ngb showed higher than Hif‐1α. In the yak's hippocampus, growth hormones such as age, sex and stress require a significant level of oxygen for these changes to occur. Ngb significant expression has a protective function as these changes take place in the hippocampus and influence adult yak behavior. However; Hif‐1α increases the level of Ngb expression as the hippocampus demands oxygen and influences the Ngb function during oxygen transport. In the hippocampus, it is observed that Ngb can decrease for long days after physiological changes but increase after a few days (Brayn et al., 2012). As suggested by the current researchers that Hif‐1α is responsible for activating Ngb in few days. Hif‐1α has been expressed in the rat hippocampus, and the administration of rAAV‐HIF‐1α also induced robust and prolonged Hif‐1α production in the rat hippocampus (Chai et al., 2014). The significant level of Hif‐1α in the hippocampus of adult yak may have the potential for attenuating hippocampal neuronal during apoptosis.

### 4.3 Amygdala

The amygdala is recognized as a component of the limbic system and is thought to play important roles in emotion and behavior. In the mouse brain, Ngb revealed its highest concentration in the amygdala and other regions (Hundahl et al., 2010), while Reuss et al. reported a considerable level of Ngb mRNA expression in the amygdala of the rodent brain (Reuss et al., 2002). Similarly, the current result found a significant level of Ngb and Hif‐1α in the amygdala of adult yak. The Ngb expression level was recorded higher than Hif‐1α. The behavior changes and other functions in the adult yak, such as protecting young adults from predators, long‐distance movement at high‐altitude, mitochondrial dysfunctions and neurodegenerative disorders require strong protection of the neuron tissues from damage. The significant Ngb expression plays an important role during these changes and functions. Meanwhile, Hif‐1α upregulates Ngb expression during its protective functions. Rob et al. suggested that a decreased level of Hif‐1α in the amygdala of adult mice has a neuroprotective function (Rob et al., 2005). These results confirmed Hif‐1α lower expression as compared to Ngb has a strong neuroprotective role in the amygdala of adult yak.

### 4.4 Olfactory lobe

The olfactory lobe is a neutral structure of the vertebrate forebrain involved in olfaction or a sense of smell. A study conducted by Chenggang et al. reported that Ngb mRNA was distributed in the olfactory lobe, and it was suggested that Ngb is a conserved gene in evolution and is very important in the nervous system (Chenggang et al., 2002). The present results showed a significant level of Ngb and Hif‐1α in the olfactory bulb of adult yak and the expression contribute to essential neuronal senses. A significant level of oxygen is needed when the yak is covering a long distance and the breath rate increase as the yak moves. The Ngb expression in the olfactory bulb regulates the oxygen rate as the yak breaths. Here, the odor concentration often increases, and the sweat gland becomes active as perspiration is taking place. The significant expression of Hif‐1α mediates Ngb expression as it plays a neuroprotective role in the olfactory bulb. The response to oxygen stimuli depends on the ability to successfully adapt to hypoxic. The pattern of Ngb expression facilitates oxygen movement between neural tissues and provides a level of neuronal protection during hypoxia. Hif‐1α expression upregulates the Ngb expression during facilitation and also aids in protecting neuronal tissues. Longbo et al. confirmed that Hif‐1α expression was observed in the olfactory bulb of newborn mice, and the expression levels contribute to developing neurons under normal conditions and hyperactive mTORC1 conditions (Longbo et al., 2016).

### 4.5 Basal ganglia

Basal nuclei function in body movement and coordination. In a 26‐year‐old male, Ngb is highly expressed in the basal nuclei while a female of 42 years showed low expression (Hu et al., 2017). Age factors may have an influence on the expression of Ngb in neuronal tissues especially the basal ganglia. The current findings reported a significant Ngb and Hif‐1α expression in the basal nuclei of the adult yak. The expression pattern may participate in protecting neuron tissues during transportation or movement in the high altitude environment. During transportation, the breath rate of yak often increases, and oxygen is paramount in this process. The significant expression of Ngb is involved in protecting the movement and coordination of neuronal tissues in the basal ganglia. The Hif‐1α expression protects the basal ganglia from hypoxic or ischaemic conditions, potentially limiting brain damage. Ngb lacks conserved hypoxia‐responsive elements (HREs) for transcriptional activation (Hif‐1α contains HREs) but contains conserved hypoxia‐inducible mRNA stabilization signals (Wystub et al., 2004). So, in the basal ganglia of adult yak, Hif‐1α expression activates Ngb functions during movement and reacts against other tumors in the body of adult yak. A study has confirmed that the strong expression of Hif‐1α in the basal nuclei and other neuron tissues reacts against tumors (Hawa et al., 2016).

### 4.6 Thalamus

Thalamus plays a function in the motor or sensory signals including visual, audio, emotion, memory and pains. The present findings recorded that Ngb and Hif‐1α expression in the adult yak thalamus have shown a significant rate of expression. However, the Ngb expression was reported higher than Hif‐1α. During sensory signals in the adult yak, Ngb regulates oxygen expression and plays a neuroprotective function. The level of Hif‐1α increases Ngb expression as it regulates oxygen in neuronal tissues. Activities such as responding to predators can be stressful and require a significant level of oxygen. Adult yaks respond to predators by huddling closely together, with the yaks on the outside of the circle lowering their horns as if ready to attack. Yaks will also try to scare predators away by charging and protecting their calves. Ngb not only responds to the oxygen demand but protects neuron tissues from damage. It is recorded that Ngb expression in the yak, mouse and murine thalamus read similarly (Della‐Valle et al., 2010; Reuss et al., 2002). Although researches have a focus on Ngb expression in the thalamus, there exist limited references.

### 4.7 Hypothalamus

The hypothalamus plays a crucial role in many important functions, including releasing hormones. regulating body temperature, heart rate, preventing brain injury and blood pressure. The current results showed a higher Ngb and Hif‐1α expression in the hypothalamus but no significant. Ngb expression in the hypothalamus promotes neuronal survival (Eliana et al., 2016). Christian et al. reported that Ngb was highly expressed in the hypothalamus of humans (Christian et al., 2013) while in the adult mouse brain, Ngb expression showed the highest but without significance (Fabrizius et al., 2016); and Hif‐1α was predominately expressed in the human hypothalamus as reported by Joana et al. (Joana et al., 2018). The high expression of Ngb in the adult yak hypothalamus is suggested to aid in supply oxygen to the blood flow in the body and acts as endogenous protectants in the nerve cells while Hif‐1α in the hypothalamus can have an oxygen‐independent regulation such as oxidative stress and because the hypothalamus is located at the base of the forebrain and around the walls of the third ventricle which received signals from the periphery through the bloodstream (Catrina, 2014; Cramer et al., 2003). Ngb expression in the adult yak hypothalamus may also be involved in preventing an imbalance in the blood flow and nutrients such as glucose and lactate, leading to biochemical and molecular changes that cause neuronal damage and Hif‐1α might attenuate Ngb functions in the hypothalamus. As confirmed by Brunori et al., Ngb has shown to involve in NO metabolism by detoxification of harmful NO under normoxic conditions (Brunori et al., 2005).

### 4.8 Cerebellum

The cerebellum receives information from the sensory systems (spinal cord) and coordinates voluntary movements such as posture, balance, coordination and speech, resulting in smooth and balanced muscular activity. In the adult mouse brain, Ngb was expressed in the cerebellum as confirmed by qRT‐PCR and Western blotting. Kunlin et al. found that Ngb expression was the highest in the cerebellum (Kunlin et al., 2010) and similar to the results of Burmester et al. (Burmester et al., 2000) who also study the human brain. The current findings revealed a significant level of Ngb and Hif‐1α expression in the adult yak cerebellum but the strength of Ngb showed higher than Hif‐1α. The presence of Ngb in the cerebellum of adult yak indicates that it plays a crucial role in the maintenance of neural cell activities and maintains stable oxygen flow while information has been integrated by the cerebellum. The Hif‐1α expression promotes and upregulates Ngb as it maintains accurate oxygen flow. Previous studies suggested that Ngb has a consensus sequence for Hif‐1α in its promotion (Brayn et al., 2012; Liu et al., 2012), although the mechanism is not fully elucidated.

### 4.9 Frontal lobe

The frontal lobe controls cognitions such as emotion, body language and reproduction. In the brain of a rat, Ngb expression was observed in the frontal lobe and their expression was measured and detected by enzyme‐linked immunosorbent assay and Western blot (Liu et al., 2012). Fabrizius et al. reported that Ngb expression was also found in the frontal lobe of the human brain (Fabrizius et al., 2016). Similarly, the current researchers recorded a significant level of Ngb and Hif‐1α expression in the adult yak frontal lobe, but the trend and strength of distribution of Ngb showed higher than Hif‐1α. The Ngb expression in the frontal lobe has a remarkable function during reproduction. During birth delivery, the adult female yak showed a highly neuroprotective maternal effect, and the presence of Ngb protects neuron cells from damage. Meanwhile, Hif‐1α might enhance O_2_ supply to Ngb and prevent cognitive impairment. In the study of Chen et al, Hif‐1α is expressed in the frontal lobe of the rat model and apoptosis in the frontal cortex. The expression is shown to protect against cognitive impairment induced by chronic cerebral ischaemic injury through an anti‐apoptotic mechanism (Chen et al., 2013).

### 4.10 Corpus callosum

Corpus callosum aids in the sensory integration between the cerebral cortex. In the current study, a significant level of Ngb and Hif‐1α was intensively expressed in the corpus callosum of the adult yak. but Ngb intensity revealed higher as compare to Hif‐1α. Ngb expression in the corpus callosum is involved in signaling transmission across the left and right hemispheres of the adult yak. Hif‐1α present in the corpus callosum increase Ngb levels in the neuron tissues as transmission occurs across the hemispheres and protects the brain from oxidative stress and severe injury. A previous study conducted by Avivi et al. reported that Ngb was observed in the corpus callosum of the subterranean mole rat (Spalax) (Avivi et al., 2010), while intense Ngb‐IR was expressed in the corpus callosum of the transgenic mice (Raida et al., 2013).

## 5 CONCLUSION

Although few references about Ngb and Hif‐1α expression are available, none of them have reported the two proteins expression in the telencephalon of adult yak. Ngb plays an important physiological role in oxygen absorption, usage and transportation of oxygen in neuronal cells and serves as an oxygen sensor to regulate signal transmission according to changes in oxygen concentration. Ngb also facilitates oxygen movement between neuron tissues and provides a secondary level of neuronal protection from hypoxia. Meanwhile, the Hif‐1α regulates and increases Ngb expression in the telencephalon and contains HREs which Ngb lacks to transcript activation. The researchers suggested that the rates of Ngb and Hif‐1α can influence the adaptive potential of adult yak to the high‐altitude environment. The current reports provided relevant data to understand Ngb and Hif‐1α influence on the adaptive mechanism and recommend further studies to explore the adaptive mechanism.

## 6 SUPPLEMENTARY MATERIALS

The analyzed data are provided in full in the results section of this manuscript and raw data deposited online (Doi.10.6084/m9.figshare.12952016).

## CONFLICT OF INTEREST

The authors declare that they have no known competing financial interests or personal relationships that could have appeared to influence the work reported in this paper.

## AUTHOR CONTRIBUTIONS

*Conceptualization*: Xiaohua Du and James Blackar Mawolo. *Data curation*: James Blackar Mawolo, Xiaoyu Mi, Qiao Li and Yongqiang Wen. *Formal analysis*: Xiaohua Du and James Blackar Mawolo. *Investigation*: Xia Liu, Xiaoyu Mi and James Blackar Mawolo. *Methodology*: Xiaohua Du, Xia Liu and Xiaoyu Mi. *Writing original draft*: Xiaohua Du and James Blackar Mawolo. *Writing‐ review and editing*: Xiaohua Du, Xia Liu, James Blackar Mawolo, Xiaoyu Mi, Qiao Li and Yongqiang Wen.

## ANIMALS AND SETTING

The Animal Ethics and Welfare Committee of Gansu Agricultural University in October of 2019 (AEWC‐GAU‐2019‐042) reviewed and approved all experimental procedures performed in this study. All animals were housed in a full facility at the cooperative city of Gannan Tibetan Autonomous Prefecture in Gansu Province of China. Three (3) healthy adult yaks at the age of 3 years were purchased from the center. The animals were housed and monitored by trained personnel and fed on grasses and sedges, such as Carex, Stipa and Kobresia. In the plateau environment of Gannan Tibetan Autonomous Prefecture, the altitude was 3000 m. Experiments were carried out using adult yak weighing 550–720 kg. The weight ranges from 10 to 15 μm. The animals were maintained at a temperature between ‐7° C and ‐8° C and had free access to food and water. Every effort was applied to reduce the number of animals used and minimize animal suffering during the sampling process.

### PEER REVIEW

The peer review history for this article is available at https://publons.com/publon/10.1002/vms3.553
